# Mutational analysis of the *Aspergillus* ambient pH receptor PalH underscores its potential as a target for antifungal compounds

**DOI:** 10.1111/mmi.13438

**Published:** 2016-07-15

**Authors:** Daniel Lucena‐Agell, América Hervás‐Aguilar, Tatiana Múnera‐Huertas, Olga Pougovkina, Joanna Rudnicka, Antonio Galindo, Joan Tilburn, Herbert N. Arst, Miguel A. Peñalva

**Affiliations:** ^1^ Department of Cellular and Molecular BiologyCentro de Investigaciones Biológicas CSICRamiro de Maeztu 9Madrid28040Spain; ^2^ Section of Microbiology, Imperial College LondonFlowers Building, Armstrong RoadLondonSW7 2AZUK

## Abstract

The *pal/RIM* ambient pH signalling pathway is crucial for the ability of pathogenic fungi to infect hosts. The *Aspergillus nidulans* 7‐TMD receptor PalH senses alkaline pH, subsequently facilitating ubiquitination of the arrestin PalF. Ubiquitinated PalF triggers downstream signalling events. The mechanism(s) by which PalH transduces the alkaline pH signal to PalF is poorly understood. We show that PalH is phosphorylated in a signal dependent manner, resembling mammalian GPCRs, although PalH phosphorylation, in contrast to mammalian GPCRs, is arrestin dependent. A genetic screen revealed that an ambient‐exposed region comprising the extracellular loop connecting TM4‐TM5 and ambient‐proximal residues within TM5 is required for signalling. In contrast, substitution by alanines of four aromatic residues within TM6 and TM7 results in a weak ‘constitutive’ activation of the pathway. Our data support the hypothesis that PalH mechanistically resembles mammalian GPCRs that signal via arrestins, such that the relative positions of individual helices within the heptahelical bundle determines the Pro316‐dependent transition between inactive and active PalH conformations, governed by an ambient‐exposed region including critical Tyr259 that potentially represents an agonist binding site. These findings open the possibility of screening for agonist compounds stabilizing the inactive conformation of PalH, which might act as antifungal drugs against ascomycetes.

## Introduction

Regulation of gene expression by ambient pH, a transcriptional response that tailors the biosynthesis of extracellular enzymes, permeases and exported metabolites to the needs imposed by the pH of the growth medium, crucially contributes to the ability of many fungi to thrive in disparate pH environments. The molecular wiring of the pH regulatory circuit has been extensively studied in the filamentous fungus *Aspergillus nidulans* and in yeasts, particularly in *Saccharomyces cerevisiae*. It includes a dedicated signal transduction pathway, denoted the *pal/RIM* pathway, that triggers, with assistance of the endosomal sorting complex required for transport (ESCRT) machinery, the proteolytic activation of the executive transcription factor PacC/Rim101p in response to alkaline pH (Peñalva and Arst, 2002; 2004; Peñalva *et al*., 2008; 2014).

As fungal pathogens invading mammalian hosts are exposed to systemic pH values above neutrality, it is unsurprising that the *pal/RIM* pathway has been identified as an important factor determining pathogenicity (Davis, 2009; Cornet and Gaillardin, 2014). Since the pioneering studies revealing the involvement of this pathway in systemic candidiasis (Davis *et al*., 2000), studies with ascomycetes as diverse as the Saccharomycotina *Candida albicans* (Nobile *et al*., 2008) and the Pezizomycotina *A. nidulans* (Bignell *et al*., 2005) and *A. fumigatus* (Bertuzzi *et al*., 2014) have uncovered its key involvement in the ability of these fungi to infect mammalian hosts. Overwhelming evidence indicates that, in ascomycetes, the *pal/RIM* pathway ambient pH sensor is a transmembrane protein denoted PalH in *A. nidulans* and Rim21p in *S. cerevisiae* (Herranz *et al*., 2005; Hervás‐Aguilar *et al*., 2007; Galindo *et al*., 2012; Obara *et al*., 2012; Herrador *et al*., 2015). This pH sensor is the most appealing component of the pathway to serve as druggable target, although it would be unsuitable for infections caused by the basidiomycetes, for example, *Cryptococcus neoformans*, given that although the *RIM* pathway is involved in its pathogenicity (O'Meara *et al*., 2010; 2013; 2014), PalH/Rim21p (and their associated arrestin, see below) appear to be missing in basidiomycete lineage (Cervantes‐Chávez *et al*., 2010; Ost *et al*., 2015). However, the fact that ascomycetes including pathogenic species such as *A. fumigatus*, *A. niger*, *C. albicans*, *C. glabrata*, *Penicillum marneffei* and *Histoplasma capsulatum* account for a large share of fungal human infections makes the possibility of targeting PalH potentially very useful.

The direct or indirect activation of this PalH sensor by ambient pH is transmitted to the internal leaflet of the plasma membrane by a transducer module involving an arrestin‐like protein, denoted PalF in *A. nidulans* and Rim8p in *S. cerevisiae*, an ubiquitin ligase (Rsp5p in *S. cerevisiae*) and the ESCRT‐I protein Vps23. PalF becomes ubiquitinated in a PalH‐dependent manner following exposure of cells to alkaline pH (Herranz *et al*., 2005), and this PalF ubiquitination triggers downstream signalling events (Hervás‐Aguilar *et al*., 2010). This occurs because ubiquitinated PalF/Rim8 in turn recruits Vps23 to plasma membrane‐associated signalling foci, sparking ESCRT‐III polymerization in these locales, which most likely serves as an amplification step (Herrador *et al*., 2009; Galindo *et al*., 2012; Obara and Kihara, 2014) (see (Peñalva *et al*., 2014) for a review). ESCRT‐III recruits to these foci the downstream *pal/RIM* pathway components PalC/Ygr122w (Rim23) (Galindo *et al*., 2007; Rothfels *et al*., 2005), PalA/Rim20p (which serves as adaptor for PacC/Rim101p (Xu and Mitchell, 2001; Vincent *et al*., 2003)) and the calpain‐like protease PalB/Rim13p (Rodríguez‐Galán *et al*., 2009; Obara and Kihara, 2014; Lucena‐Agell *et al*., 2015), which results in the proteolytic processing activation of the transcription factor (Peñalva *et al*., 2014). It is now agreed that despite the link of pH signalling to endosomal protein complexes, the pathway functions at the plasma membrane and does not require endocytosis (Calcagno‐Pizarelli *et al*., 2011; Galindo *et al*., 2012; Obara and Kihara, 2014; Lucena‐Agell *et al*., 2015).

Suggestively, PalH/Rim21p shares features with members of the seven transmembrane protein family of receptors (7‐TMRs). 7‐TMRs, usually denoted G protein‐coupled receptors (GPCRs), are the ‘most druggable’ family of proteins for pharmacological intervention (30% of the top‐selling prescription drugs are directed against GPCRs (Chalmers and Behan, 2002)), in part due to the fact that small molecules are able to target them with high specificity and affinity. Therefore, should the mechanism of PalH activation resemble that of its mammalian counterparts, the possibility of targeting the fungal ambient pH receptor with molecules interfering with its normal activity appears feasible, beside being theoretically attractive.

GPCRs do not solely transduce signals by way of heterotrimeric G proteins. Arrestins are a class of proteins previously thought to act exclusively to desensitize stimulated GPCRs. However, when engaged to the cytosolic tails of stimulated receptors, ‘classical’ β‐arrestins can recruit, activate and scaffold downstream signalling complexes without involvement of heterotrimeric G‐proteins, thus playing positive roles in signalling (Lefkowitz and Shenoy, 2005; Violin and Lefkowitz, 2007). Unlike β‐arrestins, α‐arrestins, a subfamily including the fungal clade, characteristically contain docking sites for the Rsp5 ubiquitin ligase (Alvarez, 2008) and indeed many of them act as adaptors targeting integral membrane protein partners for ubiquitin‐mediated endocytic down‐regulation (Lin *et al*., 2008; Nikko and Pelham, 2009; Karachaliou *et al*., 2013). In marked contrast, the α‐arrestin PalF/Rim8p engaging the PalH/Rim21p receptor acts positively, apparently by mediating its own requisite ubiquitination (Herrador *et al*., 2009; Herrador *et al*., 2015). PalF is necessary and, if artificially ubiquitinated, sufficient for the alkaline pH‐dependent activation of the *pal* pathway (Hervás‐Aguilar *et al*., 2010; Galindo *et al*., 2012). However, how under normal circumstances PalH/Rim21 stimulation results in PalF/Rim8p ubiquitination is currently unknown.

GPCR receptors consist of a bundle of helices embedded in the membrane, connected by cytosolic and external loops. Agonist binding to an inactive receptor R promotes a conformational transition R > R* resulting in subtle movements of the TMs that expose the otherwise inaccessible G protein‐binding sites (Sprang, 2011; Katritch *et al*., 2013). In contrast, the so denoted inverse agonists displace the equilibrium towards the inactive R state, thereby suppressing basal (i.e. in the absence of agonist) activity. However, this model is an oversimplification as receptors can adopt more than two conformations in response to different ligands, a fact that gave rise to the concept of ‘biased ligands’, i.e. ligands that specifically stabilize one particular receptor conformation. This concept is ultimately translated in that, for example, a ‘biased agonist’ elicits only a subset of the full repertoire of activities of any given receptor (Violin and Lefkowitz, 2007). Thus for receptors that can signal by either G proteins or arrestins, certain agonists are biased towards arrestin signalling, strongly supporting the view that ‘arrestin‐specific conformations’ do exist (Wisler *et al*., 2007).

**Figure 1:**
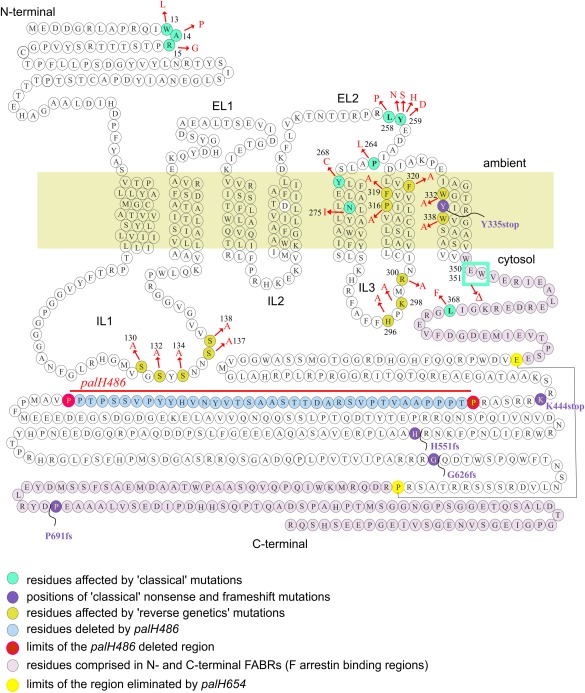
Scheme of PalH. The positions of the predicted TM domains and connecting loops relative to the lipid bilayer (olive green) are depicted. Amino acid substitutions corresponding to ‘classical’ and engineered missense mutations, the positions of truncations by ‘classical’ nonsense or frameshift alleles, the PalF arrestin binding regions (FABRs) in the cytosolic tail and the residues and borders of regions removed by deletions designed for this work are indicated.

*A. nidulans* PalH consisting of an N‐terminal integral membrane moiety (residues 1–352) and a long C‐terminal tail (residues 353–760) (Fig. 1) not only engages an arrestin, but it appears to conform to the topology of 7‐TMRs. According to TMpred (http://www.ch.embnet.org) the membrane moiety consists of seven TMs, with TM‐1 crossing the membrane in the outside‐in direction, such that its long C‐terminal tail is exposed to the cytosol. This tail contains two PalF binding regions (Fig. 1, pink residues) one adjacent to TM‐7 (residues 349–386) and the second bound by C‐terminal residues 657‐760 (Herranz *et al*., 2005).

Here, we exploit the amenability of *A. nidulans* to classical and reverse genetic analysis to obtain evidence that PalH is indeed a *pal* pathway receptor that upon exposure to alkaline pH undergoes a conformational change similar to those experienced by mammalian GPCRs, and that this change is transmitted to the cytosolic tail to transduce the alkaline pH signal by way of arrestin activation. Given that pH signalling does not involve G‐proteins, PalH/Rim21p would be a prototypical example of a class of receptors naturally biased towards arrestin signalling.

## Results

### Gene replacement procedure for PalH analysis

*A. nidulans* is haploid, facilitating the analysis of PalH function genetically, using a gene replacement procedure. We first constructed a *pyrG89 palH*Δ strain in which the *palH* coding region had been substituted by *A. fumigatus pyroA* (*pyroA^Af^*). This substitution/recombination event was confirmed by Southern blotting and phenotype testing. This *palH*Δ*::pyroA^Af^* strain was used as recipient of gene‐replacement linear DNA fragments consisting of wild‐type or mutant HA3‐tagged *palH* alleles and *A. fumigatus pyrG* as selective marker (Supporting Information Fig. 1). Transformants in which *palH* had been reconstituted with mutant versions were identified by their pyridoxine auxotrophy resulting from the exchange of *pyroA^Af^* by *palH‐HA3::pyrG^Af^* cassettes. Diagnostic plate tests for pH regulation demonstrated that strains carrying a wild‐type *palH‐HA3* gene‐replaced allele were indistinguishable from the authentic wild‐type, and western blot (WB) analyses following a shift from acidic to alkaline conditions revealed that the proteolytic activation processing patterns of PacC in *palH‐HA3* and *palH^+^* strains were indistinguishable (Supporting Information Fig. 1), thereby showing that the genetic manipulations do not by themselves impair PalH function.

### PalH is phosphorylated in an ambient pH‐ and PalF‐dependent manner

**Figure 2:**
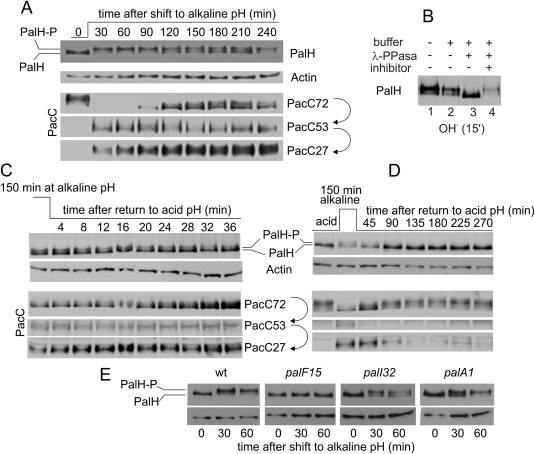
PalH phosphorylation in alkaline pH conditions. A. Top, time course of PalH phosphorylation upon shifting cells from acidic to alkaline pH conditions for the indicated times. PalH‐P is phosphorylated PalH. Bottom, time‐course of PacC processing in the same cells. In all PacC western blots the ‘empty’ gel regions located between PacC72 and PacC53 and between the latter and PacC27 are not shown. Actin serves as loading control. B. The two bands of PalH with different mobility coalesce into a single faster moving band after lambda bacteriophage phosphatase treatment (samples from cells shifted to alkaline pH for 15 min). C. Time course of PalH dephosphorylation in cells that had been shifted from acidic to alkaline pH for 150 min and then back to acidic conditions for the indicated times. The bottom panel shows the corresponding anti‐PacC western blot. The relative levels of PacC53 and PacC27 change little over the time scale of this experiment, whereas PacC72 accumulates gradually, reflecting the fact that the pathway is inactive. D. A similar time course experiment in which cells were sampled between 45 min and 4.5 h after shifting them down to acidic pH. Note how PacC27 gradually disappears due to degradation and the absence of signalling proteolysis, combined with transcriptional repression of the *pacC* promoter mediated by PacC72 (Bussink *et al*., 2015). E. PalH phosphorylation is PalI‐ and PalA‐independent and PalF‐dependent. *palF15*, *palI32* and *palA1* are phenotypically null mutations in the corresponding genes.

The pH signalling pathway responds to alkaline pH. Under acidic conditions the transcription factor PacC is not proteolytically activated, being present as the primary translation product, PacC72. Alkalinization of the medium activates the *pal* pathway, triggering the proteolytic activation of PacC72 to PacC27 via the committed intermediate PacC53 (Díez *et al*., 2002) (anti‐PacC western blots, Fig. 2A). In anti‐HA3 WBs of these cells cultured under acidic conditions PalH appears as a single band. However, PalH is modified upon exposing cells to alkalinity, such that an additional band(s) of reduced mobility become(s) the predominating species (Fig. 2A). This reduction in the mobility of PalH was reversed by treatment with lambda phosphatase (Fig. 2B), indicating that the mobility shift(s) result(s) from phosphorylation.

PalH remained phosphorylated in cells kept at alkaline pH for as long as 4 h (Fig. 2A). When alkaline‐exposed cells were shifted back to acidic pH, the relative level of phosphorylated species declined slowly, correlating with the ‘recovery’ of PacC72 caused by the absence of signalling (i.e. the lack of proteolytic processing of freshly synthesized PacC) (Fig. 2C). Complete dephosphorylation took as long as 45–90 min after returning cells to acidic pH, a time at which the pool of PacC27, which turns over also slowly (Mingot *et al*., 1999), becomes barely detectable (note that PacC72 does not increase further because transcriptional autorepression counteracts the absence of its proteolytic processing (Bussink *et al*., 2015)) (Fig. 2D). All the above data are consistent with PalH becoming accessible to phosphorylation as a consequence of alkaline pH sensing. The correlation between pathway activation and PalH phosphorylation was suggestive as upon their agonist‐mediated activation mammalian GPCRs are rapidly phosphorylated by GRKs (G protein receptor kinases), hampering signalling (Shukla *et al*., 2011; Lefkowitz, 2013). Notably, although PalH phosphorylation is independent of PalI (a PalH helper) and of PalA (acting downstream of ESCRT recruitment), it is strictly dependent on PalF (Fig. 2E), indicating that PalH phosphorylation requires the functional engagement of the arrestin to the receptor.

### Mutant PalH with a cytosolic tail restricted to the two PalF arrestin binding sites retains some function

**Figure 3:**
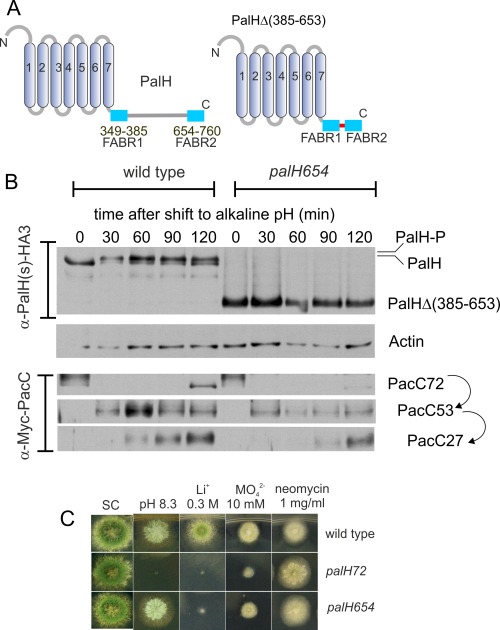
mutant PalH with a cytosolic tail containing just the arrestin binding regions (FABRs) retains substantial function. A. Scheme of the mutant *palH654* product compared to the wild‐type. B. Time course of PalH phosphorylation and PacC processing in pH shift experiments with *palH654* and wild‐type cells. C. Diagnostic growth tests of pH signalling in the indicated strains. Note that although almost unable to grow on plates containing Li^+^, *palH654* grows like the wild‐type at pH 8.3, a pH at which the null *palH72* strain is unable to grow. *palH654* also displays a noticeably stronger resistance to molybdate than *palH72*, although lesser than the wild‐type.

Equipped with the gene replacement procedure, we first constructed an allele encoding a PalH protein in which cytosolic residues 385 through 653 separating the two PalF binding sites had been substituted by a synthetic linker consisting of a Gly‐Ala pentamer (*palH654*, Fig. 3A). WB showed that the protein is stably expressed (Fig. 3B). Null *palH* alleles such as *palH72* truncating PalH after residue 12, or alleles truncating PalH within the TM domain do not grow at alkaline pH, display exacerbated sensitivity to Li^+^ and ${\text{MoO}}_{4}^{2-}$ ion toxicity and enhanced resistance to neomycin (Negrete‐Urtasun *et al*., 1999; Herranz *et al*., 2005) (Fig. 3C). Remarkably, *palH654* strains are, like *palH72* strains, highly sensitive to lithium. Yet, unlike *palH72*, *palH654* strains grow at alkaline pH and are more resistant than *palH72* strains to molybdate, showing that the mutant *palH654* product is, to a significant extent, functional. WB analysis of PacC processing buttressed this conclusion. In pH shift experiments the null *palH72* mutation blocked the proteolytic processing activation of PacC (Hervás‐Aguilar *et al*., 2010). In contrast, *palH654* impaired it relatively weakly (Fig. 3B, bottom), although this allele led to a reduction in the overall PacC levels. We conclude that although the 269 cytosolic residues of PalH located between the previously delimited PalF binding regions play some role, this region is not critical for pH signalling.

### Phosphorylation is largely dispensable for PalH function

Anti‐HA3 WBs of *palH654* cells strongly suggested that the phosphorylation‐mediated shift in PalH mobility requires amino acids located within the large 269 residue region deleted in the mutant (Fig. 3B). This large segment of the protein might contain phosphoacceptor residues or it might be required indirectly, for example if it contributes a docking site for the phosphorylating kinase(s). To distinguish between these possibilities, we set out to identify phosphorylatable residues throughout the protein. We first ruled out that a region containing five Ser residues between positions 130 and 138, within the first intracellular loop (IL1) connecting TM1 and TM2 (Fig. 1) serves as phosphoacceptor. We generated *palH138* encoding a mutant PalH with Ala substitutions for all five of these serines. WBs revealed normal PalH phosphorylation, eliminating these serine residues as phosphoacceptors (data not shown). In passing, *palH138* behaved as wt in diagnostic tests of pH regulation and showed a normal pattern of PacC72 processing in response to alkaline pH, indicating that these serines are not functionally/structurally important (data not shown).

**Figure 4:**
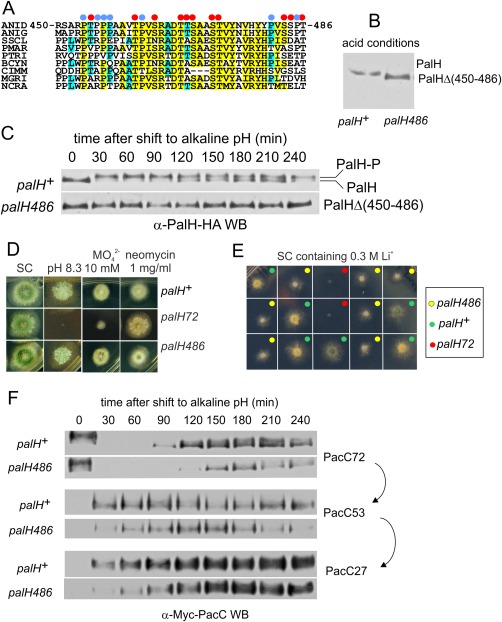
A conserved region rich in Pro, Ser and Thr residues is required for PalH phosphorylation. A. Multiple sequence alignment showing conservation of the PalH cytosolic tail region located between resides 450 and 486. Blue and red dots indicate Pro and Ser/Thr residues, respectively, in *A. nidulans* PalH. Amino acid highlighting in yellow and blue indicates strong and weak conservation, respectively. ANID, *A. nidulans*; ANIG, *A. niger*; SSCL, *Sclerotinia sclerotium*; PMAR, *Penicillium marneffei*; PTRI, *Pyrenophora tritici‐repentis*; BCYN, *Botrytis cinerea*; CIMM, *Coccidioides immitis*; MGRI, *Magnaporthe grisea*; NCRA, *Neurospora crassa*. B. Western blot analysis of equally loaded samples showing slightly elevated levels of the *palH486* mutant product. C. pH shift experiment showing the apparent absence of alkaline pH‐induced phosphorylation (as determined by changes in SDS‐PAGE mobility) of the *palH486* mutant product. D. *palH486* is indistinguishable from the wild‐type on pH 8.3, molybdate and neomycin media. E. *palH486* is hypersensitive to Li^+^. Different clones of a cross between *palH486* and *palH^+^* parental strains were point inoculated onto medium containing 0.3 M Li^+^. Yellow and green dots indicate strains that had been genotyped as *palH486* and *palH* ^+^, respectively. Red dots indicate a *palH72* strain included as control. Yellow and green surface *palH^+^* colonies reflect the fact that the yellow spore marker *yA2* is segregating in the cross (wild‐type spores are green; spores are not visible in *palH486* clones due their impaired growth on lithium medium). F. Comparison of the PacC proteolytic processing activation pattern of *palH486* with the wild‐type during a pH shift experiment. Note the slightly delayed processing and the slower increase in PacC72 in the mutant after long times at alkaline pH.

In view of this, we focused on a highly conserved region located between Pro450 and Pro486, rich in Pro, Thr and Ser residues (Fig. 4A). We generated *palH486* encoding a mutant PalH deleted for this 37‐residue motif. *palH486* had four phenotypic effects. First, it resulted in an increase, albeit very modest, in the steady state levels of PalH (Fig. 4B). Second, the mutant receptor did not undergo any changes in mobility upon shifting cells to alkaline pH, showing that the deleted region is required for phosphorylation (Fig. 4C). Third, although *palH486* strains behave essentially as the wild‐type in alkaline pH, molybdate and neomycin sensitivity growth tests (Fig. 4D), they display weak hypersensitivity to lithium chloride (Fig. 4E). Amongst 48 progeny obtained from a backcross between a *palH486* strain and the wild‐type 20 were phenotypically wild‐type and 28 were lithium hypersensitive. According to a psi‐square test these figures are not significantly different from those expected for a single Mendelian character distribution. Six wild‐type and six lithium hypersensitive clones showed the expected PCR amplification bands for wild‐type *palH* and *palH486*, respectively, indicating that lithium hypersensitivity co‐segregates with the *palH486* deletion, and thus that the phenotype is unlikely to be caused by a secondary mutation. Lastly, analysis of PacC processing showed that *palH486* causes a slight delay in the activation of the pathway within 2 h after shifting cells to alkaline conditions (Fig. 4F), a defect which appears insufficient to cause detectable effects in diagnostic tests of pH regulation (with the exception of the highly sensitive Li^+^ toxicity test), perhaps because these growth tests are scored after a 48 h incubation. Thus phosphorylation (i.e.: those phosphorylation events detectable by mobility shift in SDS‐PAGE) is largely dispensable for PalH function.

**Figure 5:**
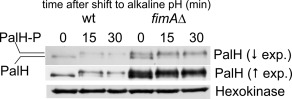
iImpairment of endocytosis results in phosphorylated PalH under acidic conditions and elevates the levels of the receptor. Western blot analysis of PalH in a null fimbrin (*fimA*Δ) mutant compared to the wild‐type. Two different exposures of same the blot are shown. Hexokinase was used as loading control.

We thus considered the possibility that PalH phosphorylation is required for the turnover of the receptor by endocytosis. To test this, we used *fimA*Δ ablating the endocytic patch component fimbrin, which results in a major defect in endocytosis (Upadhyay and Shaw, 2008). Figure 5 shows that in sharp contrast with the wild‐type, a proportion of phosphorylated PalH is detectable in *fimA*Δ cells under acidic conditions, strongly indicating that *fimA*Δ stabilizes this form. As *fimA*Δ prevents the endocytic internalization of PalH‐GFP (Lucena‐Agell *et al*., 2015), this build‐up of the phosphorylated form suggests that phosphorylation tags the protein for endocytic turnover, a possibility that is consistent with the increase in the steady state levels of PalH detected in *fimAΔ* (and in *palH654*) cells.

### Investigating PalH function by site directed mutagenesis

**Figure 6:**
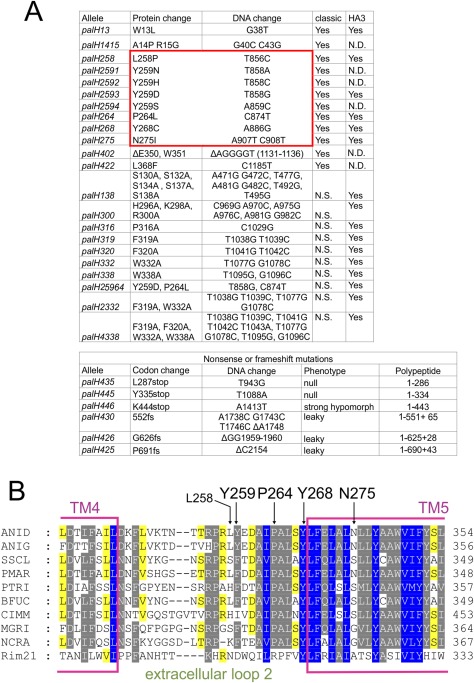
Mutational analysis of PalH: a potential agonist binding site. A. Complete catalogue of mutations reported here (with the exception of internal deletion alleles described in the text and Fig. 1). For missense mutations (top), ‘classic’ and HA3 indicate whether the allele was obtained by UV or site‐directed mutagenesis, respectively (the latter were all HA3‐tagged). N.D. is ‘not done’ whereas N.S. is ‘not selected’. The red line boxes those missense mutations that we hypothesize comprise an agonist binding site. For nonsense/frameshifting mutations (bottom) their phenotypes are indicated. Note that mutant PalH proteins encoded by leaky truncating alleles contain the TM domain‐proximal PalF binding region (Herranz *et al*., 2005), which explains their partial loss‐of‐function phenotype. *palH45* and *palH47*, described by Negrete‐Urtasun *et al*. (1999) and Herranz *et al*. (2005) are also in this category. B. Multiple sequence alignment in the PalH region containing missense mutations hypothetically representing an agonist binding site. Species are as in Fig. 4 with the exception of *S. cerevisiae*, whose Rim21p is included to underline the remarkable sequence conservation existing between filamentous fungi and budding yeast homologues in TM5.

To gain further insight into the mechanisms of alkaline ambient pH sensing, we performed a genetic screen using the gamma amino‐butyrate (GABA) technique. Briefly, *areA^r^* mutants that cannot utilize nitrogen sources other than ammonium are unable to grow on GABA as nitrogen source unless the pH signalling pathway is switched off, because the GABA permease gene is repressible by PacC27, the end product of the PacC proteolytic activation cascade (Caddick *et al*., 1986; Hutchings *et al*., 1999; Espeso and Arst, 2000). To select pH signal‐impairing mutations specifically mapping to *palH* we exploited the procedure of Tilburn *et al*. (2005), screening for GABA utilizers among mutagenized spores of a diploid strain homozygous for *areA^r^* and heterozygous for a null mutation in *palH*. Besides less informative early truncating mutations, which are not discussed for simplicity, eleven novel *palH* missense mutations resulting in partial loss‐of‐function were identified by DNA sequencing (Figs. 1 and 6). A twelfth mutation, *palH402*, is a two‐codon deletion.

*palH402* removing E350 and W351 results in complete loss‐of‐function. These residues belong to Trp^349^‐Glu^350^‐Trp^351^ motif located in the interface between the cytosol‐proximal C‐terminus of TM7 and the cytosolic tail (Fig. 1). The functional importance of this motif is supported by its strong conservation in ascomycetes, including PalH/Rim21 receptors from organisms as distant from filamentous ascomycetes as *S. cerevisiae* and *Yarrowia lipolytica* (Tréton *et al*., 2000). The Trp‐Glu‐Trp motif is contained within a 36 residue sequence (residues 349–385 of the PalH cytosolic tail) which is sufficient to interact with PalF in two‐hybrid assays (Herranz *et al*., 2005), strongly indicating that this motif plays a key role in receptor‐arrestin binding/coupling. Another mutation almost certainly impairing PalF binding to PalH is *palH422*, which affects the conserved Leu368 also located within the 36‐residue N‐terminal (i.e. membrane‐proximal) PalF binding site in the cytosolic tail. This position cannot tolerate Phe. Substitution by Ala of adjacent Gly369 debilitates the interaction with PalF (Herranz *et al*., 2005). The isolation of these mutations validated the efficacy of the genetic screen.

Among the remaining 10 missense mutations, two, *palH13* and *palH1415* affect residues located in the N‐terminal extracellular tail, indicating that this region of the protein has a functional role. Remarkably, eight of these mapped to a region encompassing the 23 residue‐long extracellular loop connecting TM4 with TM5 and the N‐terminal half (the ‘outside’ half) of TM5. Notable among these are those affecting Leu258 and Tyr259, located within a conserved motif of this loop. Tyr259 (Phe tolerated in other fungi) is highly conserved (Fig. 6B). Four different substitutions (to Asn, His, Ser, Asp) involving this residue resulted in partial loss‐of‐function, indicating that Tyr259 plays an important physiological role. Pro264Leu also affects this TM4‐TM5 loop, whereas Tyr268Cys affects the loop‐TM5 interface. Both Pro264 and Tyr268 are conserved. Lastly Asn275Ile affects a residue located in the extracellular space‐proximal half of TM5. In other PalH orthologues, a polar residue generally occupies the position of PalH Asn275. These polar residues must be protected from the lipid environment within the 3D structure, arguably by a compensating charge that would become ‘unpaired’ if position 275 were occupied by a purely aliphatic side chain residue. In any case this genetic analysis strongly indicates that residues within both TM5 and the loop connecting it with TM4 are important for PalH activity.

### PalH phosphorylation can be uncoupled from PalH activation

We next examined the effects that hypomorphic *palH* alleles isolated in the GABA screen have on the proteolytic activation of PacC. We generated *palH13* (W13L), *palH258* (L258P), *palH2593* (Y259D), *palH264* (P264L), *palH268* (Y268C) and *palH275* (N275I) HA3‐tagged alleles by gene replacement. All six prevented growth on 0.3 M Li^+^ plates and resulted in hypersensitivity to molybdate albeit to a lesser extent than a *palH72* null (Supporting Information Fig. 2), consistent with their causing partial loss‐of‐function.

**Figure 7:**
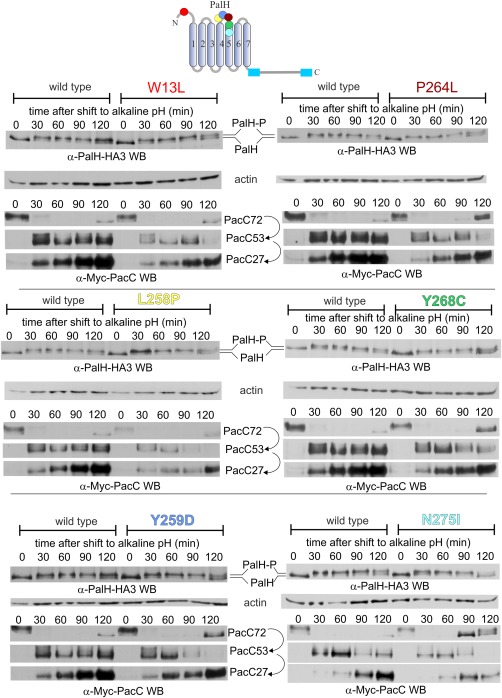
Molecular characterization of single missense mutants affecting extracellular residues. Time courses of PalH phosphorylation and PacC processing in pH shift experiments with cells carrying single residue substitutions in residues exposed to the ambient. The colours of the dots indicating mutated residues in the PalH scheme correspond with those of the different panel labels. Mutations are denoted by the resulting substitutions, in single letter amino acid code (see Fig. 6 for standard allele nomenclature and corresponding DNA changes).

Y259D, P264L, Y268C and, more conspicuously, N275I affected the persistence of signalling, as noted by the abnormal accumulation of unprocessed PacC72 at the 120 min time points (and at the 90 min time point in the case of N275I) (Fig. 7). All of these led to a reduction in PacC levels, in some cases very noticeably, possibly reflecting that the steady state levels of PacC are reduced when PacC proteolytic activation is impaired, as a consequence of the negative auto‐regulatory loop that determines the steady‐state situation (Bussink *et al*., 2015). Notably, none of these mutations decreased PalH levels (Fig. 7, anti‐HA blots), and thus their effects can only be attributable to PalH dysfunction. Thus, these data confirm the inferences derived from growth tests, establishing that the above missense mutations impair pH signalling.

We next considered the possibility that, as in mammalian GPCRs, PalH phosphorylation occurred upon receptor stimulation. However, all of the mutant PalH proteins showed normal *in vivo* phosphorylation upon a shift to alkaline pH (Fig. 7), indicating that PalH phosphorylation does not correlate with receptor function. However, virtually all the above mutations led to lower levels of the PalH phosphorylated form at the 120 min time point, hinting at some degree of connection between reduced PalH function and the inability of these mutant PalH proteins to maintain the phosphorylated state.

**Figure 8:**
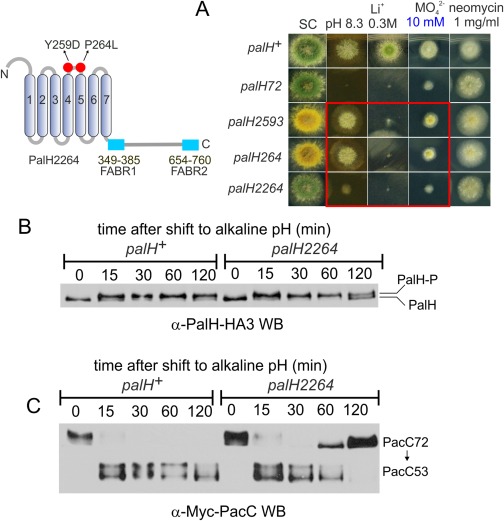
Phenotypic additivity of Y259D and P264L: molecular characterization. A. Diagnostic growth tests showing phenotypic additivity between *palH2593* (Y259D) and *palH264* (P264L). *palH2264* encodes a doubly‐substituted PalH. *palH72* is a null *palH* mutation. B. The time courses of alkaline pH‐induced wild‐type and Y259D, P264L double mutant PalH phosphorylation are very similar. C. Effects of the double mutant substitution on the signalling proteolysis of PacC72; (Note that for emphasis only the region of the gel containing PacC72 and PacC53 is included).

In view of the above, we considered the possibility that the extent to which the above missense mutations impair signalling is insufficient to be reflected in the steady‐state levels of phosphorylation. Thus, we constructed the *palH2264* allele encoding a double mutant protein combining Y259D and P264L. Unlike either parental, the double mutant was as sensitive to molybdate as the null and showed markedly decreased growth at alkaline pH, indicating additivity (Fig. 8A). The double substitution did not affect the steady state PalH levels nor the alkaline pH‐induced phosphorylation of PalH beyond the reduction in relative levels of phosphorylated form observed in the respective single mutants at the 120 min time point (Fig. 8B; for comparison see Fig. 7). By anti‐PacC WB, *palH2264* led to an abnormal accumulation of PacC72 already at 60 min after an alkaline pH shift, which was even more striking at the 120 min time point (Fig. 8C). Thus the synthetic negative effect in pH signalling displayed by these two mutations does not correlate with decreased PalH phosphorylation, indicating that PalH phosphorylation cannot solely reflect the activation of the pathway.

### Gain‐of‐function mutations in PalH obtained by site‐directed mutagenesis

Studies with GPCRs have uncovered the ‘molecular switches’, which are interactions between residues located within the heptahelical bundle that stabilize the inactive conformation of the receptor and that are disrupted after ligand binding (Xie and Chowdhury, 2013) (Schwartz *et al*., 2006). At the cytoplasmic side of GPCRs, activation results in conformational changes of the helical bundle generally involving shifts in the positions of helices V, VI and VII (Katritch *et al*., 2013). In the β2‐adrenergic receptor (β2‐AR) both TM5 and TM6 contain one Pro residue each that introduce kinks in the helices. Agonist binding modifies these kinks' angles, causing a swing in the cytosolic part of TM6 that exposes receptor epitopes to downstream transducers (Rasmussen *et al*., 2011; Rosenbaum *et al*., 2011; Warne *et al*., 2011). In certain types of GPCRs aromatic residues located within TM6 and TM7 modulate the basal activity of the receptor through the bend angle kink (Shi *et al*., 2002; Xie and Chowdhury, 2013).

**Figure 9:**
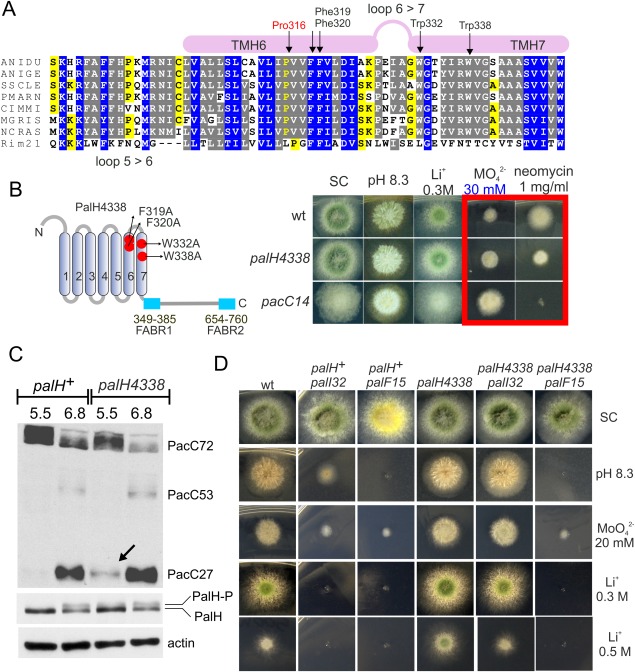
Characterization of *palH4338* resulting in pH signalling pathway activation at acidic pH. A. Multiple sequence alignment showing the region corresponding to TM6 and TM7, as in Fig. 6 (Rim21 is from *S. cerevisiae*). The positions of residues that were mutated to investigate the hypothetical involvement in alkaline pH signalling of conformational rearrangements of the helical bundle of transmembrane helices are indicated. B. Diagnostic growth tests of pH signalling in which *palH4338* is compared to the wild‐type and to a strong alkalinity‐mimicking *pacC^c^*14 allele. Note the intermediate response of *palH4338* on molybdate and neomycin plates (boxed in red). C. *palH4338* results in weak processing of PacC72 at acidic pH (pH 5.5) (the arrow indicates PacC27, the end product of the proteolytic cascade). Note the faint degree of PalH phosphorylation at pH 5.5 seen with the mutant but not with the wild‐type. Cells were cultured overnight at ‘constant’ pH before proceeding to total protein extraction followed by anti‐PacC (Myc3) and anti‐PalH (HA3) western blotting. Actin was used as loading control. D. Diagnostic tests of pH regulation showing that *palF15* is epistatic to *palH4338* whereas *palI32* is hypostatic.

Inspired by these studies we addressed, by site directed mutagenesis of TM6 and TM7 residues, the possibility that PalH undergoes conformational changes resulting in shifts in the positions of the TMs decodable by the arrestin module. TM6 contains a proline residue, Pro316, located within a conserved 316‐PVVFF‐320 motif (Fig. 9A), suggesting that, by analogy to GPCRs, this Pro may kink the helix, and that the nearby Phe residues might regulate the bending angle. Thus in a first approach we substituted Pro316 by Ala. This allele (*palH316*, Fig. 6) resulted in weak hypersensitivity to Li^+^ and ${\text{MoO}}_{4}^{2-}$ ions and increased tolerance to neomycin, and impaired PacC processing markedly, indicating loss‐of‐function (Supporting Information Fig. 3). However, these phenotypic characteristics are attributable to the rapid and marked decrease in this mutant PalH levels at alkaline pH (Supporting Information Fig. 3). Encouraged by such pH‐induced instability, arguably consistent with a requirement for this Pro in pH‐mediated conformational rearrangements involving TM6, we undertook further mutagenesis studies aimed at ‘releasing’ a hypothetical molecular switch maintaining PalH in an inactive conformation. We focused on a potential aromatic toggle involving TM6 and TM7 residues, substituting by Ala four conserved aromatic amino acids including Phe319 and Phe320 in the proximity of Pro316 in TM6 and Trp332 and Trp338 in TM7 (Fig. 9B). The quadruple substitution allele was denoted *palH4338*. Remarkably *palH4338* strains showed hyper‐resistance to both Li^+^ and ${\text{MoO}}_{4}^{2-}$ and hypersensitivity to neomycin (Fig. 9B), strongly indicating that the quadruple substitution activates the pathway. We next established rigorously that these gain‐of‐function phenotypes result from *palH4338* after outcrossing the mutant and confirming that they segregated like a single Mendelian character linked to the mutant allele, genotyped by PCR (data not shown). Furthermore, as *palH* is tightly linked to the cloramphenicol resistance gene *camC*, we demonstrated that the molybdate resistance phenotype associated with *palH4338* is closely linked to *camC* (no recombinants obtained in *n* = 49 progeny of a *camC108* x *palH4338* heterozygous cross), largely ruling out the possibility that it is caused by an unrelated mutation.

Thus, we studied the effect of *palH4338* in PacC processing. Instead of monitoring 2 h time courses after shifting cells to alkaline pH we used overnight cultures adjusted to acidic or neutral pH to approximate the steady‐state conditions of (neutral pH) plate tests. In the wild‐type cultured under neutral conditions PacC appeared as a mixture of PacC72, PacC53 and PacC27 (Fig. 9C). In contrast, under acidic conditions wild type cells contained only PacC72, in sheer contrast with *palH4338* cells, which contained detectable levels of PacC27, indicative of pathway activation (Fig. 9C). Thus, PacC processing data agree with the *palH4338* gain‐of‐function phenotype. During the course of these experiments we also noticed that at pH 5.5 *palH4338* cells but not *palH^+^* cells display a faint band of phosphorylated PalH (Fig. 9C). This observation indicates that the quadruple substitution indeed affects the basal conformation of the receptor, and might suggest that such conformational change facilitates, under acidic pH conditions, the engagement of the PalF arrestin in a manner conductive to PalH phosphorylation [which is alkaline pH‐ and PalF‐dependent (Fig. 2E)]

Next, we performed epistasis analysis. *palF15* and *palI32* are null mutations in the genes encoding the PalF arrestin‐like and the PalI ‘helper’, respectively (PalI is a protein acting upstream of PalH assisting its plasma membrane localization (Calcagno‐Pizarelli *et al*., 2007); the localization of PalI to the plasma membrane is PalH‐independent, see Supporting Information Fig. 4). *palF15* prevents growth at pH 8.3, whereas *palI32* allows some growth at this pH because the absence of PalI does not impede *pal* signalling completely. We crossed *palH4338* into null *palI32* and *palF15* backgrounds. Notably a double *palH4338 palI32* mutant grew like the wild‐type, i.e. much better than the single *palI32* parental mutant, at alkaline pH (Fig. 9D), confirming that *palH4338* has a signal‐promoting effect even without PalI. In contrast *palH4338* did not rescue to any extent the inability of *palF15* strains to grow at alkaline pH, demonstrating that the pH signalling activity resulting from *palH4338* requires PalF. Similar results were obtained with molybdate or lithium toxicity tests, where *palH4338* suppressed the hypersensitivity phenotype resulting from *palI32* but not that resulting from *palF15* (Fig. 9D). Thus, all the above data strongly support the contention that *palH4338* abnormally stimulates pH signalling under acidic conditions by promoting an arrestin‐activating conformation of PalH.

### Contribution of individual substitutions in palH4338 to gain‐of‐function

**Figure 10:**
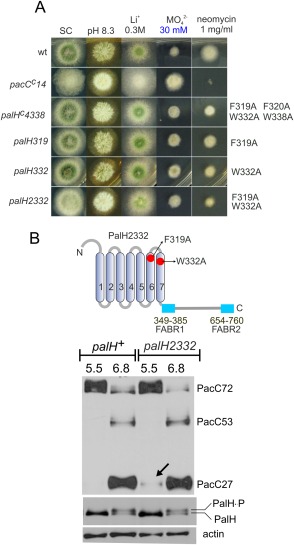
Phe319 and Trp332 seem important to maintain the basal activity of PalH. A. Diagnostic growth tests of pH regulation in the indicated strains. B. Scheme showing the positions of Phe319 and Trp332 in PalH. C. Effects of the double mutant substitution on the proteolytic processing of PacC72 in the steady state situation at the indicated pH; Note the weak activation of the pathway as indicated by detection of a faint band of PacC27.

In an attempt to identify the individual contributions of the residues mediating the effects of *palH4338* we constructed four alleles each containing one of its four missense mutations. In plate tests Phe319Ala and Trp332Ala led to weak gain of function (Fig. 10A), whereas Phe320Ala led to weak loss‐of‐function and Trp338Ala behaved as wild‐type (data not shown). Thus, we combined Phe319Ala with Trp332Ala in *palH2332* (Fig. 10A and B). PacC processing assays showed that *palH2332* extracts displayed detectable bands of PacC27 in acidic conditions that were not seen in wild‐type controls (Fig. 10B). The double substitution resulted in slightly higher neomycin sensitivity than its constituents, suggesting gain of function, but did not increase ${\text{MoO}}_{4}^{2-}$ tolerance any further, showing that its activating effect is weak (Fig. 10A). Thus, Phe319 and Trp332 in TM6 and TM7, respectively, may contribute to maintain PalH in the basal conformation, and these combined with the above data suggest that the relative position of the helices within the TM bundle participates in pH signal reception, with possible involvement of not only Phe319 in TM6 but also of Trp332 in TM7. This is very suggestive given that in the β2‐AR arrestin‐biased ligands preferentially affect the conformational states of TM7 (Liu *et al*., 2012).

## Discussion

**Figure 11:**
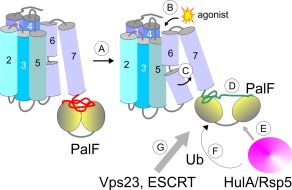
Hypothetical model for the transduction of the alkaline ambient pH signal in *A. nidulans* PalH alternates between basal and active conformations. Unlike β−arrestins PalF is bound to the receptor even under acidic (i.e. non‐signalling) conditions which drive the receptor towards the basal conformation (A). (B) Alkalization of the medium generates an ‘agonist’, perhaps a lipid of the external leaflet of the plasma membrane, that upon change of its ionization status binds a region located at the ambient‐exposed interface between TM4 and TM5. Agonist binding drives the equilibrium towards the active conformation. We speculate that the transition from the basal to the active conformation results from an alkaline pH‐ and TM6 Pro316‐dependent shift in the orientation of TM6 and TM7, a shift that is also promoted by *palH^c^4338* independently of ambient pH (C). This shift would be then transmitted to arrestin‐binding regions in the cytosolic PalH tail (D), leading to a conformational change that facilitates the recruitment of the HulA/Rsp5 ubiquitin ligase (E) and the subsequent ubiquitination of PalF (F). Next ubiquitinated PalF recruits ESCRT‐I Vps23 (G), which primes ESCRT‐III polymerization that, we hypothesize, serves as a signal amplification step because the remaining Pal proteins acting downstream of PalF (PalA, PalC and PalB) are all ESCRT‐III interactors that are transiently localized to punctate structures at the plasma membrane when ambient pH is alkalinized.

PalH is the ascomycete alkaline pH signal sensor and PalF is the key component of a signal‐transducing module also involving ESCRT‐I Vps23, which is recruited to pH signalling complexes by ubiquitinated PalF/Rim8 (Herrador *et al*., 2009; Hervás‐Aguilar *et al*., 2010; Galindo *et al*., 2012; Obara and Kihara, 2014). In all likelihood Vps23 triggers an amplification step that involves ESCRT‐III polymerization, thus creating multiple docking sites for the downstream' *pal/RIM* signalling components PalC/Ygr122w/Rim23, PalA/Rim20 and PalB/Rim13 and, by way of its interaction with PalA/Rim20, for PacC72/Rim101 (Fig. 11) (Vincent *et al*., 2003; Xu *et al*., 2004; Galindo *et al*., 2007; Herrador *et al*., 2009; Rodríguez‐Galán *et al*., 2009; Calcagno‐Pizarelli *et al*., 2011; Galindo *et al*., 2012; Obara and Kihara, 2014; Peñalva et al., 2014; Herrador *et al*., 2015; Lucena‐Agell *et al*., 2015). Vps4‐mediated ESCRT‐III de‐polymerization terminates signalling (Galindo *et al*., 2012). However, despite this detailed understanding of the pathway, the mechanistic bases of pH signal reception (i.e. the modification(s) that PalH undergoes in response to alkaline pH) and transduction (i.e., how are these conformational changes in the PalH TM receptor result in PalF ubiquitination) are insufficiently understood.

This work provides two important novel pieces of information when considered in the light of extensive knowledge available on the mechanisms of 7‐TMR activation. One is that an extracellular region comprising the TM4‐TM5 extracellular loop and residues in the ambient‐proximal half of TM5 are required for signalling. By analogy to mammalian GPCRs (Katritch *et al*., 2013), this region might contribute to an agonist binding site. The second is that aromatic residues embedded within TM6 and TM7 are necessary to maintain PalH in an inactive state, as their substitution by alanines weakly activates the pathway. An attractive interpretation of this observation is that TM6/TM7 substitutions result in rearrangements of the heptahelical bundle that are transmitted to PalF, promoting its transition from the basal to the active conformation. PalF activation results in ubiquitination of the arrestin (Herranz *et al*., 2005). Upon binding to cognate active receptors mammalian arrestins undergo conformational changes (Hirsch *et al*., 1999; Shukla *et al*., 2013), which expose otherwise masked epitopes to effectors. One such PalF effector must be the NEDD4 ubiquitin ligase (Rsp5 in *S. cerevisiae*) (Herrador *et al*., 2009), denoted HulA in *A. nidulans* (Karachaliou *et al*., 2013)). As overwhelming evidence indicates that Vps23 binds only to ubiquitinated PalF (Galindo *et al*., 2012), the hypothetical conformational change that PalF undergoes would be the actual transducer of the alkaline pH signal.

In both two‐hybrid assays and pull‐downs using a bacterially expressed GST‐PalH tail bait, PalF and the cytosolic tail of PalH are strong interactors (Herranz *et al*., 2005; Galindo *et al*., 2012), indicating that binding of the arrestin to the receptor does not require phosphorylation of the latter, contrary to the situation reported for mammalian GPCR/arrestin complexes. In agreement, a PalH mutant deficient in phosphorylation is largely functional (Fig. 4). Thus, even though our results cannot rule out a contribution of PalH phosphorylation to arrestin binding, this contribution would be largely inconsequential. Co‐expression experiments *in vivo* indicated that PalF binds to PalH even under acidic conditions (Hervás‐Aguilar *et al*., 2010), and Rim21p has been shown to interact with Rim8p in a ubiquitin split assay on standard yeast media (Herrador *et al*., 2015), supporting a scenario in which the PalH/PalF complex is preformed *in vivo*, irrespective of the pH conditions. Strikingly, the cytosolic tail (without the TMs) of yeast Rim21p (Rim21CT) localizes in part to the PM (Herrador *et al*., 2015), which might help to position Rim8 (yeast PalF) in this locale. The intrinsically unstable active conformation of GPCRs is stabilized by G protein binding at the cytosolic side of the membrane (Sprang, 2011). PalF/Rim8 binding to PalH/Rim21 might reduce the energy required by the receptor to acquire the signalling conformation, thereby increasing its basal activity (i.e. the probability that any one PalH receptor at any one moment is signalling under acidic conditions). This would ensure that there is some ‘housekeeping’ activity of the pathway under acidic circumstances. Such interpretation is consistent with the observation that PalF overexpression results in weak activation of the pathway (Hervás‐Aguilar *et al*., 2010).

Transduction of the alkaline pH signal essentially requires, besides the TM region of PalH, the two PalF binding sites present in its cytosolic tail (Fig. 1). The one adjacent to TM7 contains 12 acidic (Asp/Glu) residues out of a total 36. Thus a highly speculative possibility is that this region acts as a phosphomimetic epitope, playing the role of the GRK‐phosphorylated residues in mammalian GPCRs. Direct signal‐independent engagement of PalF to this region might facilitate the transmission of structural rearrangements in PalH to the PalF fold. According to our mutagenesis analysis aromatic residues in the highly conserved PalH TM7 might be implicated in structural rearrangements, which is a very suggestive finding in view of the fact that conformational changes in TM7 have been implicated in arrestin‐biased signalling by GPCRs (Wacker *et al*., 2013)

A major unresolved question is how is alkaline pH sensed by PalH/Rim21, including whether it is directly activated or not by alkaline pH. A potentially important observation was that the *RIM* pathway is activated by altering lipid asymmetry (Ikeda *et al*., 2008), raising the intriguing possibility that the receptor actually senses a lipid whose ionization status or whose localization changes rapidly in response to alkalinity. It has been proposed that the ability of the C‐terminal cytosolic moiety of Rim21p (Rim21CT) to bind the PM is itself modulated by pH‐dependent changes in lipid asymmetry, implying that the cytosolic region may contribute to signal reception (Nishino *et al*., 2015).

A GPCR receptor responding to the lipid sphingosine‐1‐phosphate has been characterized structurally (Hanson *et al*., 2012). Here the lipid ligand gains access to its pocket laterally, within the transmembrane moiety of the receptor, because access to the extracellular milieu from the top is blocked by the amino terminus and extracellular loops. This situation would be consistent with our genetic data, such that Trp13 and Ala14/Arg15 in the amino terminal region together with Leu258, Tyr259 and Pro264 in EL2 could form the ‘roof’ of a lipid pocket embedded in the outer leaflet of the plasma membrane and contribute to stabilizing agonist binding, thereby explaining why these residues are required for signalling. A model involving cooperation between the N‐terminal external domain and a ligand binding site contributed by the transmembrane moiety has been proposed to explain the regulation of the 7‐TM receptor Smoothened, the key transducer of the metazoan Hedgehog pathway (Rana *et al*., 2013). We have previously noted the similarities between the *pal/RIM* and Hedgehog pathways (Arst and Peñalva, 2003), similarities further highlighted by the subsequent seminal finding that β‐arrestin 2 mediates Smoothened signalling (Chen *et al*., 2004). It is worth emphasizing that Tyr259 within the EL2 in PalH appears to be critical. Theoretically, there are six possible missense mutations resulting from single nucleotide substitution within the TAT Tyr259 codon. Four, involving substitutions of Tyr259 by Asn, His, Asp and Ser were isolated as partial loss‐of‐function mutations, suggesting that rather than any ionization status of Tyr259 it is the aromatic moiety which is physiologically important for receptor activation.

Herrador *et al*. have recently reported that the activity of yeast Rim8 (PalF) is antagonized by CK1‐mediated, Rim21‐dependent phosphorylation (Herrador et al., 2015). We have shown that PalH is phosphorylated in alkaline pH‐ and PalF‐dependent manner, raising the possibility that the same kinase that is recruited by the activated arrestin phosphorylates the receptor. However, PalH phosphorylation does not seem to have any important role in promoting signalling. An instructive finding was that despite the fact that phosphorylation is normally seen only when the pathway is activated, phosphorylated PalH is detectable at acidic pH in a mutant deficient in endocytosis, indicating that the cells preferentially turnover phosphorylated PalH as compared to the non‐phosphorylated pool. Thus, we speculate that phosphorylation contributes to maintaining the steady‐state levels of PalH at the plasma membrane.

In summary, our results are consistent with a mechanism of PalH activation bearing resemblance to that of mammalian 7‐TM receptors: a ‘pocket’ accommodates an unidentified ‘ligand’ that triggers a shift in the relative positions of individual TM helices within the helical bundle. Therefore our data strongly support the possibility that PalH is a druggable target, potentially alleviating the devastating impacts of ascomycete fungi not only in animal and plant pathogenesis, but also in toxin production, food spoilage and building material damage, all of which are subordinated to the jurisdiction of the pH signalling pathway (Peñalva and Arst, 2002, 2004; Peñalva *et al*., 2008).

## Experimental procedures

### Media and growth tests

*Aspergillus* complete (MCA) and synthetic complete medium (SC) (Cove, 1966), containing 1% glucose and, unless otherwise indicated, 5 mM ammonium tartrate as carbon and nitrogen sources, respectively, were used for growth tests and strain maintenance. Strains, which carried markers in standard use, are listed in Table 1. pH shift experiments involving transfer of cells from acidic (pH 4) to alkaline conditions (pH ∼8) have been described (Hervás‐Aguilar *et al*., 2010, 2007; Lucena‐Agell *et al*., 2015). Overnight cultures under acidic (final pH ∼5) or neutral (final pH ∼6.5) pH conditions (Hervás‐Aguilar *et al*., 2010) were used for some experiments involving *palH4338* (see text for details).

**Table 1:** Strains used in this work.

Strain	Genotype
MAD976	*yA*2 *pabaA*1 *argB*2 *palI*32
MAD1293	*fwA*1 *inoB*2 *palH*72 *argB*2::[alcAp::palI::gfp::argB*]
MAD1349	*inoB*2 *palH*72 *pacC*900
MAD1445	*yA*2 *pabaA*1 *pacC*14900
MAD1730	*pyroA*4 *inoB*2 *nkuA*Δ*::bar pacC*900
MAD1829	*inoB*2 *palI*32 *pacC*900
MAD1977	*wA*4 *pabaA*1 *pacC*900 *palF*15
MAD2336	*pantoB*100 *palH::ha_3_::pyrGfum pyrG*89*?*
MAD2341	*palI*32 *palH::ha_3_::pyrGfum pyrG*89*?*
MAD2344	*palA*1 *palH::ha_3_::pyrGfum pyrG*89*? pyroA*4
MAD2337	*palF*15 *palH::ha_3_::pyrGfum pyrG*89*? pantoB*100
MAD2668	*palH*Δ*::pyroAfum pacC*900 *pyroA*4 *pyrG*89*? nkuA*Δ*::bar*
MAD2743	*yA*2 *pyroA*4 *pantoB*100
MAD2885	*palH::ha_3_::pyrGfum pyroA*4 *pacC*900 *pyrG*89 *nkuA*Δ*::bar*
MAD2886	*palH*13*::ha_3_::pyrGfum pyroA*4 *pacC*900 *pyrG*89 *nkuA*Δ*::bar*
MAD2887	*palH*258*::ha_3_::pyrGfum pyroA*4 *pacC*900 *pyrG*89 *nkuA*Δ*::bar*
MAD2888	*palH*2593*::ha_3_::pyrGfum pyroA*4 *pacC*900 *pyrG*89 *nkuA*Δ*::bar*
MAD2889	*palH*264*::ha_3_::pyrGfum pyroA*4 *pacC*900 *pyrG*89 *nkuA*Δ*::bar*
MAD2890	*palH*268*::ha_3_::pyrGfum pyroA*4 *pacC*900 *pyrG*89 *nkuA*Δ*::bar*
MAD2891	*palH*275*::ha_3_::pyrGfum pyroA*4 *pacC*900 *pyrG*89 *nkuA*Δ*::bar*
MAD3002	*palH*486*::ha_3_::pyrGfum pyroA*4 *pacC*900 *pyrG*89 *nkuA*Δ*::bar*
MAD3319	*yA*2 *pabaA*6
MAD3877	*pyroA*4 *pacC*900
MAD3880	*palH*486*::ha_3_::pyrGfum pyrG*89*? pyroA*4 *pabaA*1 *pacC*900
MAD4256	*palH::ha_3_::pyrGfum pacC*900
MAD4258	*palH*654*::ha_3_::pyrGfum pyroA*4 *pacC*900 *pyrG*89 *nkuA*Δ*::bar*
MAD4261	*palH*4338*::ha_3_::pyrGfum pyroA*4 *pacC*900 *pyrG*89 *nkuA*Δ*::bar*
MAD4270	*fimA::Tn*431*::pyr‐4 pacC*900 *pyroA*4 *pyrG*89*? nkuA*Δ*?*
MAD4336	*palH*4338*::ha_3_::pyrGfum pyrG*89*? pyroA*4
MAD4413	*palH*316*::ha_3_::pyrGfum pyroA*4 *pacC*900 *pyrG*89 *nkuA*Δ*::bar*
MAD4416	*palH*138*::ha_3_::pyrGfum pyroA*4 *pacC*900 *pyrG*89 *nkuA*Δ*::bar*
MAD4418	*palH*300*::ha_3_::pyrGfum pyroA*4 *pacC*900 *pyrG*89 *nkuA*Δ*::bar*
MAD4421	*palH*4338*::ha_3_::pyrGfum pyroA*4 *pacC*900 *pyrG*89 *nkuA*Δ*::bar*
MAD4422	*palH*654*::ha_3_::pyrGfum pacC*900 *pyrG*89*? pyroA*4
MAD4552	*yA*2 *palH*13*::ha_3_::pyrGfum pabaA*6 *pacC*900 *pyrG*89*?*
MAD4554	*yA*2 *palH*258*::ha_3_::pyrGfum pabaA*6 *pacC*900 *pyrG*89*?*
MAD4556	*yA*2 *palH*2593*::ha_3_::pyrGfum pabaA*6 *pacC*900 *pyrG*89*?*
MAD4557	*yA*2 *palH*264*::ha_3_::pyrGfum pabaA*6 *pacC*900 *pyrG*89*?*
MAD4559	*palH*268*:: ha_3_::pyrGfum pabaA*6 *pacC*900 *pyrG*89*?*
MAD4576	*yA*2 *pantoB*100 *pyroA*4 *palF*15
MAD4685	*palH*275*::ha_3_::pyrGfum pabaA*6 *pacC*900 *pyrG*89*?*
MAD4874	*palH*319*::ha_3_::pyrGfum pyroA*4 *pacC*900 *pyrG*89 *nkuA*Δ*::bar*
MAD4876	*palH*320*::ha_3_::pyrGfum pyroA*4 *pacC*900 *pyrG*89 *nkuA*Δ*::bar*
MAD4878	*palH*332*::ha_3_::pyrGfum pyroA*4 *pacC*900 *pyrG*89 *nkuA*Δ*::bar*
MAD4880	*palH*338*::ha_3_::pyrGfum pyroA*4 *pacC*900 *pyrG*89 *nkuA*Δ*::bar*
MAD4882	*pyroA*4 *pacC*900 *palH*72
*MAD5140*	*palH*2332*::ha_3_::pyrGfum pyroA*4 *pacC*900 *pyrG*89 *nkuA*Δ*::bar*
MAD5142	*palH*25964*::ha3::pyrGfum pyroA*4 *pacC*900 *pyrG*89 *nkuA*Δ*::bar*
MAD5205	*palI*32 *palH*4338*::ha_3_::pyrGfum pyroA*4 *pacC*900 *pyrG*89? *nkuA*Δ*?*
MAD5207	*wA*2 *palH::ha_3_::pyrGfum fimA::Tn*431*::pyr‐4 pacC*900 *pyroA*4 *pyrG*89*? nkuAΔ?*
MAD5216	*yA*2 *pabaA*6 *palH*316*::ha_3_::pyrGfum pyrG*89*? pacC*900
MAD5218	*pabaA*6 *palH*138*::ha_3_::pyrGfum pyrG*89*? pacC*900
MAD5220	*yA2 palH*300*::ha_3_::pyrGfum pyroA*4 *pabaA*6 *pyrG*89*? pacC*900
MAD5224	*palH*654*::ha_3_::pyrGfum pyroA*4 *pacC*900 *pyrG*89? *nkuA*Δ*::bar*
MAD5225	*palF*15 *palH*4338*::ha_3_::pyrGfum pyroA*4 *pacC*900 *nkuA*Δ*?*

### Selection and characterization of previously undescribed palH mutations by random mutagenesis

Essentially the diploid γ‐aminobutyrate (GABA) selection method of Tilburn *et al*. (2005) was used to select new *palH* mutations. A diploid of genotype *pabaA1 yA2 aroC660 palH72 camC108*; *areA^r^−5; pantoB100/areA^r^−5; inoB2; glrA1; fwA1* was constructed by several meiotic crosses. *palH* (AN6886) is closely linked and centromere‐distal to *aroC* (AN6866) (http://www.aspgd.org/) and *camC* is even more tightly linked to *palH* (Arst *et al*., 1994). Inclusion of *aroC660* and *camC108* in coupling to *palH72* minimizes the selection of mitotic recombinant *palH72* homozygous diploids: The absence of aromatic amino acids in the selection medium virtually rules out obtaining single crossover events centromere‐proximal to *aroC660* and screening for reduction of chloramphenicol resistance in diploids eliminates most single crossover events centromere‐distal to *aroC660*. (*camC108* is partially dominant (Gunatilleke *et al*., 1975)). After UV mutagenesis, conidiospores of the above diploid were top‐layered into glucose‐minimal medium containing 5 mM GABA as sole nitrogen source and 0.054% (w/v) sodium deoxycholate and incubated for 4–5 days at 37°C. Diploids able to use GABA as nitrogen source were picked off and tested phenotypically at 37°C for sensitivity to 3.5 mg/ml chloramphenicol and GABA utilization, and at both 37°C and 20°C for growth at pH 8 and for sensitivity to 20 mM ${\text{MoO}}_{4}^{2-}$ on glucose minimal medium with 5 mM ammonium tartrate as nitrogen source. Resulting diploids impaired for growth at pH 8 and hyper‐sensitive to ${\text{MoO}}_{4}^{2-}$ toxicity were haploidized on complete medium containing benlate (Hastie, 1970) but without additional supplementation of *pabaA1* or *aroC660* and *yA^+^* colonies were purified on otherwise supplemented glucose minimal medium lacking p‐aminobenzoate and aromatic amino acids. This ensured that virtually all haploids recovered carried the *palH* allele in repulsion to *palH72*. The haploids were tested phenotypically for other markers present in the diploids along with GABA utilization, chloramphenicol and ${\text{MoO}}_{4}^{2-}$ sensitivity and growth at pH 8. As experiments to obtain *palH* mutations having a null phenotype yielded mainly frame‐shift and chain termination alleles lacking major regions of the protein (with the exception of *palH402*), we mostly focused on obtaining partial loss‐of‐function mutations in order to maximize chances of getting single residue substitutions and other phenotypically less drastic sequence changes. Phenotypically promising *palH* alleles were then sequenced.

### Site‐directed mutagenesis and construction of palH alleles by gene replacement

This procedure is graphically summarized in Supporting Information Fig. 1. A strain (MAD1737 in our collection, MAD indicating Madrid) with genotype *pyrG89*; *pyroA4 nkuA::bar*; *pacC900* [the latter encoding wild‐type Myc‐tagged PacC (Hervás‐Aguilar *et al*., 2007)] (Table 1), requiring both pyrimidines and pyridoxine for growth, was used to substitute the complete coding region of *palH* by *A. fumigatus pyroA*. The resulting strain, denoted MAD2668 (*pyrG89 palH*Δ*::pyroA^Af^*; *pyroA4 nkuA*Δ*::bar*; *pacC900*) still requires pyrimidines but it is prototrophic for pyridoxine. This strain was used as recipient for transformation with mutated *palH* alleles, all constructed after mutagenesis of plasmid p1949. This plasmid contains a DNA fragment including ∼1500 bps of *palH* 5′‐flanking region followed by the complete *palH* coding region, tagged with HA3 in the C‐terminus, followed by *A. fumigatus pyrG* (*pyrG^Af^*) as selective marker and then by ∼1500 bps of 3′‐flanking region facilitating recombination. The wild‐type and mutated versions of this fragment can be excised as a linear DNA molecules by appropriate restriction enzyme digestion and used to replace *palH*Δ*::pyroA^Af^* in MAD2668 by engineered *palH* alleles. As control we reconstructed a wild‐type *palH::HA3* allele denoted *palH806*. Strains containing this allele were indistinguishable from a ‘true’ wild‐type in diagnostic tests of pH regulation (Supporting Information Fig. 1). Mutagenesis was carried out using the Stratagene QuickChange site‐directed mutagenesis kit and mutagenic primers. All mutant plasmids were confirmed by DNA sequencing and, when appropriate (internal *palH* deletions*)*, by restriction enzyme digestion.

Gene replaced transformants selected by their pyrimidine‐independent growth were tested on plates lacking pyridoxine, as the gene replacement event made these transformants auxotrophic for pyridoxine. Clones thus selected were confirmed to carry gene‐replaced *palH* alleles by Southern blotting. A preliminary assessment of their phenotypes was carried out and, if necessary, a selected primary transformant for any given mutation was outcrossed to confirm that the phenotype segregated as a single Mendelian character, linked to the *palH::HA3* allele, whose presence was diagnosed by PCR.

### Protein extraction and western blots

With the single exception described below, Western blot analyses were made using ‘total protein samples’ obtained by a direct alkaline solubilization of lyophilized mycelium (Hervás‐Aguilar and Peñalva, 2010), which minimizes protein degradation and dephosphorylation. The sources of antibodies and the dilutions used for detection of Myc‐PacC, PalF‐HA3 (or PalH‐HA3), hexokinase and actin have been detailed previously (Lucena‐Agell *et al*., 2015). Diagnostic tests of pH regulation based on alkaline pH (pH 8.3), lithium (0.3 or 0.5 M), sodium molybdate (10, 20 or 30 mM, as required) or neomycin sulfate (1 mg/ml) sensitivity have all been described (Tilburn *et al*., 1995; Peñas *et al*., 2007; Lucena‐Agell *et al*., 2015).

To isolate membrane fractions used for lambda phosphatase treatment, lyophilized mycelia (collected from cultures shifted for 15 min at pH 8.3 to trigger phosphorylation of PalH) were ground to a fine powder with a FP120 FastPrep and a ceramic sphere. 80 mg of powdered mycelia were hydrated with 1 ml of buffer MPE (100 mM Tris HCl, 150 mM NaCl, 5 mM EDTA and 25 mM N‐ethylmaleimide, pH 7.5, containing Roche's EDTA‐free protease inhibitor cocktail) and mixed with 0.45 µm glass beads. The resulting slurry was FastPrep‐homogenized again. Large debris were pelleted by centrifugation at 3000 rpm for 3 min at 4°C in a microcentrifuge. The supernatant was spun for 45 min at 14,000 g and the resulting pellet was resuspended in 1 ml MPE, washed once in the same buffer and finally resuspended in 180 µl. Aliquots were mixed with lambda phosphatase buffer (New England Biolabs) and, where indicated, with lambda phosphatase. 10 mM sodium orthovanadate was used for the ‘plus inhibitor’ control reaction. Reactions were incubated for 20 min at 30°C before precipitating proteins with 10% TCA and proceeding with anti‐HA western blotting of the immunoprecipitates.

## Author contributions

DL‐A, AH‐A, TM‐H, OP, JR, and HNA carried out the experimental work. DL‐A, AH‐A, AG, JT, HNA and MAP designed and interpreted experiments. MAP and HNA wrote the paper.

## Supporting information

Supporting Information
