# Supplementary material for: Mutational analysis of the Aspergillus ambient pH receptor PalH underscores its potential as a target for antifungal compounds
Source: Mol Microbiol. 2016 Jul 15;101(6):982–1002. doi: 10.1111/mmi.13438 (PMC5026065; doi:10.1111/mmi.13438)
Supplement: Supplementary file 1 — Supporting Information [file MMI-101-982-s001.pdf]

Supplementary Information

Mutational analysis of the *Aspergillus* ambient pH receptor PalH underscores its potential as a target for antifungal compounds

Daniel Lucena-Agell[1], América Hervás-Aguilar[1], Tatiana Munera-Huertas[2], Olga Pougovkina[1], Joanna Rudnicka[2], Antonio Galindo[1], Joan Tilburn[2], Herbert N. Arst, Jr.[1,2], and Miguel A. Peñalva[1]
[1] Department of Cellular and Molecular Biology, Centro de Investigaciones Biológicas CSIC, Ramiro de Maeztu 9, Madrid 28040, Spain. [2] Section of Microbiology, Imperial College London, Flowers Building, Armstrong Road, London SW7 2AZ, UK.

Supplemental Figure 1
Top, scheme of the gene replacement procedure used for targeted mutagenesis of *palH*. Details may be found under experimental procedures. Middle, diagnostic growth tests of pH regulation showing that a *palH::HA3* strain cannot be distinguished from an authentic *palH*$^{+}$ wild-type. Control *palH72* and *pacC*^{c}*14* strains are included for comparison. The acidity-mimicking *palH72* mutation is a null *palH* mutation resulting in strong sensitivity to alkaline pH, lithium and molybdate ions, and in increased resistance to neomycin. The alkalinity-mimicking *pacC*^{c}*14* mutation does not affect growth at alkaline pH, increases tolerance to lithium and molybdate ions and results in high sensitivity to neomycin. Bottom, pH shift experiment showing that the proteolytic processing activation patterns of PacC72 in the wild-type and in the *palH::HA3* strains are essentially indistinguishable. The faint band that appears in all lanes above PacC72 is a non-specific protein occasionally reacting with some lots of anti-Myc antibody, used to detect PacC.

Supplemental Figure 2
Diagnostic tests of pH regulation for the engineered *palH* alleles resulting in the indicated substitutions (see Figures 6 and 7).

Supplemental Figure 3
(A) Diagnostic tests of pH regulation for *palH316* showing that it behaves as a partial loss-of-function mutation. (B) Western blots of cells shifted from acidic to alkaline conditions showing that PalH P316A appears to be degraded under alkaline conditions (top) and that the proteolytic processing pattern of PacC72 to PacC53 and PacC27 is impaired.

Supplemental Figure 4
The absence of *palH* does not affect the plasma membrane localization of PalI. Top, PalI-GFP localization in a *palH*Δ background, showing the plasma membrane fluorescence signal (in inverted contrast; the boxed region is displayed at 2.5 times magnification on the right). Bottom, in germlings and nascent germtubes PalI is strongly polarized (blue arrowheads). PalI also labels the plasma membrane of septa (red arrowheads). Thus the distribution of PalI in a *palHΔ* strain is as reported in the wild-type [Calcagno-Pizarelli AM, Negrete-Urtasun S, Denison SH, Rudnicka JD, Bussink HJ, et al. (2007) Establishment of the ambient pH signaling complex in *Aspergillus nidulans* : PalI assists plasma membrane localization of PalH. Eukaryot Cell 6: 2365-2375.]

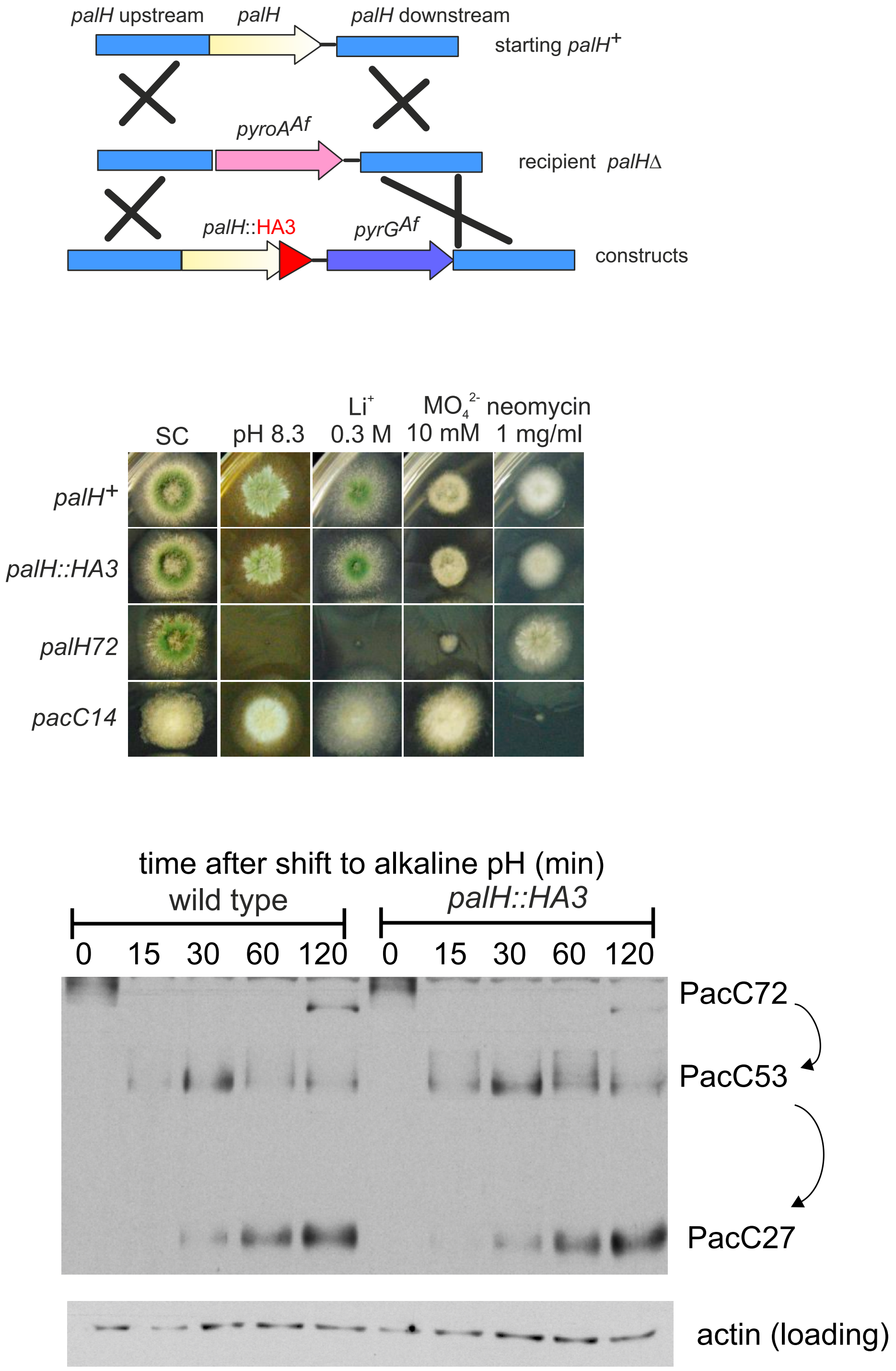

Supplemental Figure 1
Top, scheme of the gene replacement procedure used for targeted mutagenesis of *palH*. Details may be found under experimental procedures. Middle, diagnostic growth tests of pH regulation showing that a *palH::HA3* strain cannot be distinguished from an authentic *palH*$^{+}$ wild-type. Control *palH72* and *pacC*^{c}*14* strains are included for comparison. The acidity-mimicking *palH72* mutation is a null *palH* mutation resulting in strong sensitivity to alkaline pH, lithium and molybdate ions, and in increased resistance to neomycin. The alkalinity-mimicking *pacC*^{c}*14* mutation does not affect growth at alkaline pH, increases tolerance to lithium and molybdate ions and results in high sensitivity to neomycin. Bottom, pH shift experiment showing that the proteolytic processing activation patterns of PacC72 in the wild-type and in the *palH::HA3* strains are essentially indistinguishable. The faint band that appears in all lanes above PacC72 is a non-specific protein occasionally reacting with some lots of anti-Myc antibody, used to detect PacC.

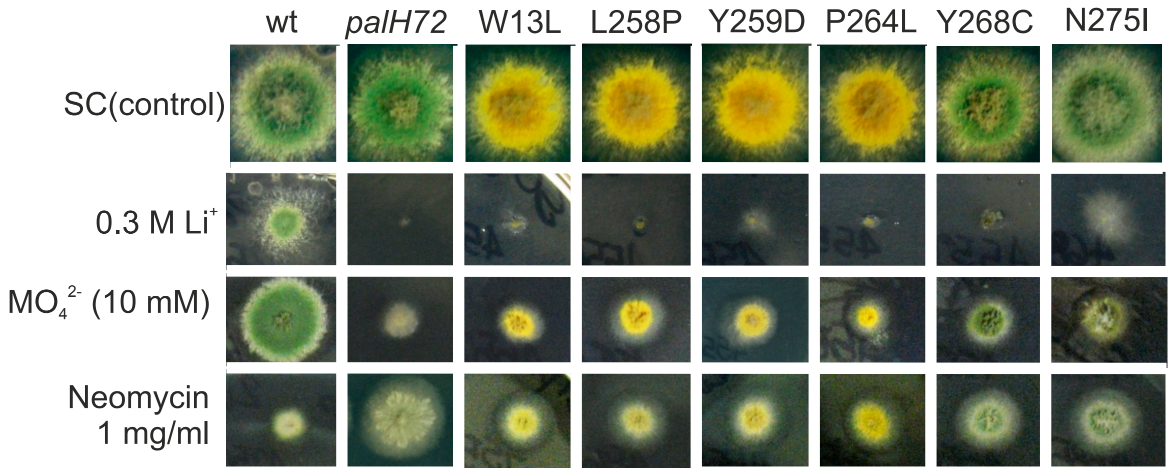

Supplemental Figure 2
Diagnostic tests of pH regulation for the engineered *palH* alleles resulting in the indicated substitutions (see Figures 6 and 7).

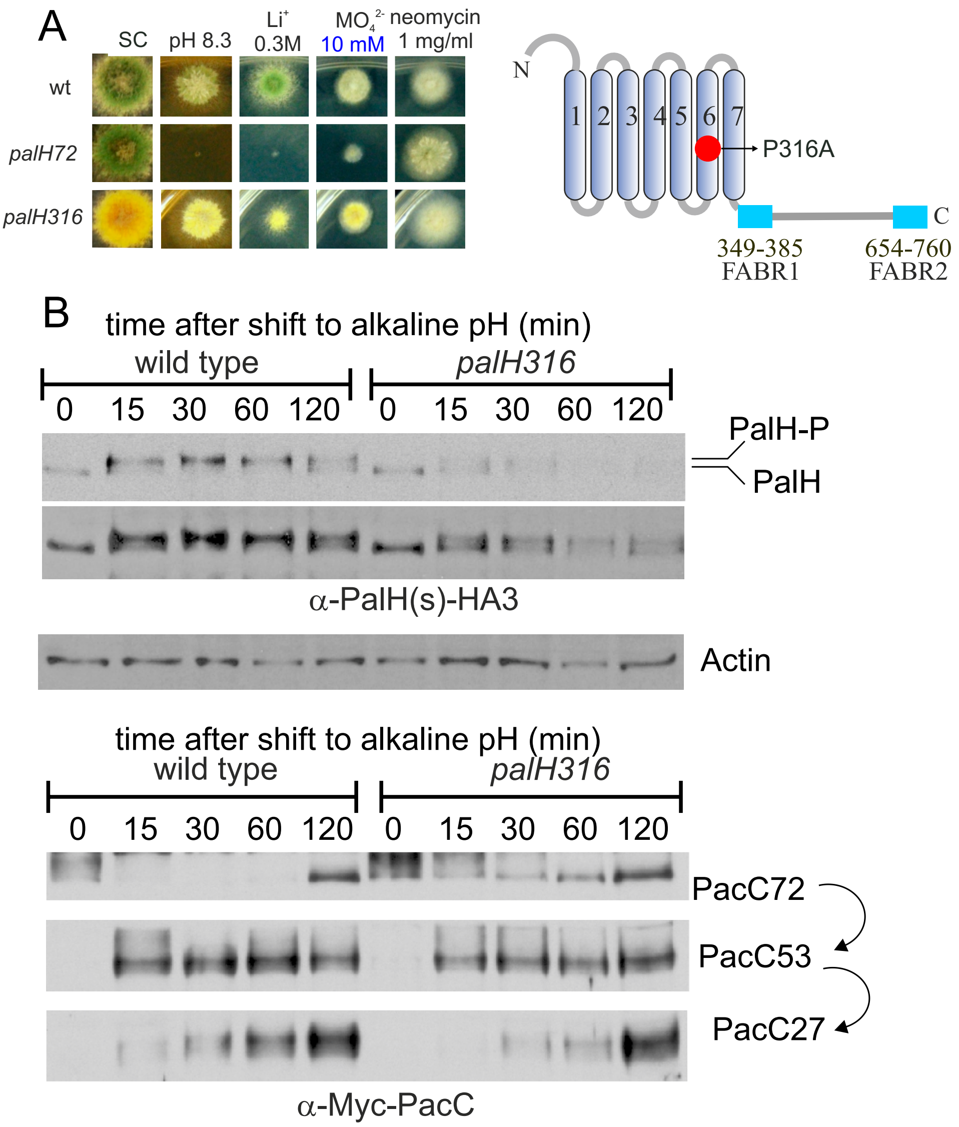

Supplemental Figure 3

(A) Diagnostic tests of pH regulation for *palH316* showing that it behaves as a partial loss-of-function mutation. (B) Western blots of cells shifted from acidic to alkaline conditions showing that PalH P316A appears to be degraded under alkaline conditions (top) and that the proteolytic processing pattern of PacC72 to PacC53 and PacC27 is impaired.

PalI-GFP in *palH*Δ

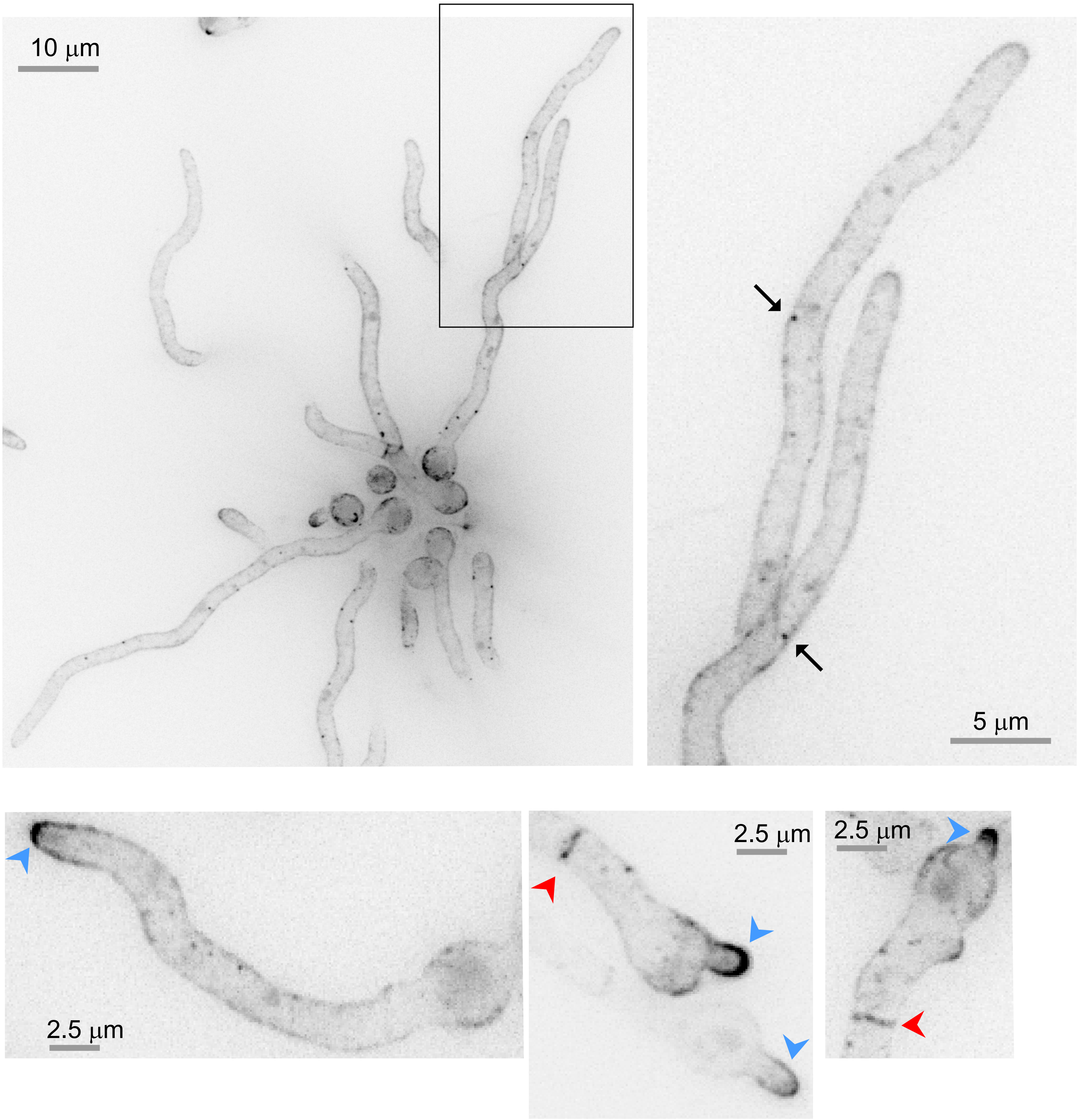

Supplemental Figure 4
The absence of *palH* does not affect the plasma membrane localization of PalI. Top, PalI-GFP localization in a *palH*Δ background, showing the plasma membrane fluorescence signal (in inverted contrast; the boxed region is displayed at 2.5 times magnification on the right). Bottom, in germlings and nascent germtubes PalI is strongly polarized (blue arrowheads). PalI also labels the plasma membrane of septa (red arrowheads). Thus the distribution of PalI in a *palH*Δ strain is as reported in the wild-type [Calcagno-Pizarelli AM, Negrete-Urtasun S, Denison SH, Rudnicka JD, Bussink HJ, et al. (2007) Establishment of the ambient pH signaling complex in *Aspergillus nidulans*: PalI assists plasma membrane localization of PalH. Eukaryot Cell 6: 2365-2375.]
